# Difficult Patient Encounters: Assessing Pediatric Residents' Communication Skills Training Needs

**DOI:** 10.7759/cureus.3340

**Published:** 2018-09-21

**Authors:** Kimberly Collins, Akshata Hopkins, Nicole A Shilkofski, Rachel B Levine, Raquel G Hernandez

**Affiliations:** 1 General Pediatrics, Johns Hopkins All Children's Hospital, Saint Petersburg, USA; 2 General Pediatrics, Johns Hopkins University School of Medicine, Baltimore, USA; 3 Internal Medicine, Johns Hopkins University School of Medicine, Baltimore, USA; 4 General Pediatrics, Johns Hopkins All Children's Hospital, Tampa, USA

**Keywords:** communication skills training, simulation, curriculum development, needs assessment, difficult patient encounters, graduate medical education, pediatric residents

## Abstract

Introduction

Difficult patient encounters (DPEs) are common and can lead to frustration and dissatisfaction among healthcare providers. Pediatric resident physician experiences with DPEs and curricula for enhancing necessary communication skills have not been well described.

Materials and methods

We used a cross-sectional survey research design for our needs assessment on resident experiences with DPEs. Thirty-three pediatric residents completed this anonymous survey. The survey assessed residents’ experiences with and self-efficacy regarding DPEs. Descriptive statistics were used to analyze the quantitative data. Additionally, two authors independently coded free response data to include in the narrative description of the survey results.

Results

These survey results include the views of 92% of the residents in the program (33/36). Residents reported a greater frequency of difficult encounters in the inpatient setting than the outpatient setting. The majority of residents rated their communication skills during DPEs as “fair” or “good” (70%, 23/33). Residents tended to have lower confidence when discussing chronic pain, managing parental insistence on a plan, and breaking bad news. They generally reported higher levels of anxiety for scenarios involving angry patients and families, families insisting on a plan, and when breaking bad news. Residents cited many challenges, including working with angry and demanding families. Additionally, residents described difficulty with managing discordant opinions between the family and the healthcare team regarding the care plan. Residents expressed a preference for learning how to manage challenging patient encounters using clinical experiences. Simulation, discussion, and observation of role models also rated highly as educational methods for increasing skills, while most residents rated lectures as the least important means of training skills for these difficult encounters.

Discussion

We found that pediatric residents experience difficult encounters frequently, especially in the inpatient setting. Individual residents vary in their confidence and anxiety levels with different types of difficult encounters and may benefit from not only general communication skills training, but also from targeted training to equip them for the particular contexts they find most challenging. Residents value interactive structured learning activities, including discussion and simulation. Residents most consistently value the opportunity to lead challenging conversations in the clinical setting, especially when followed by effective debriefing and feedback by trained faculty preceptors.

Conclusions

Next steps include creating a “Difficult Encounters” communication skills curriculum informed by this needs assessment, which aim to enhance patient care as well as increase resident self-efficacy. In addition to the curriculum development for residents, it may be helpful to initiate faculty development on how to supervise resident-led difficult conversations and provide effective debriefing and feedback to promote resident growth.

## Introduction

Difficult patient encounters

Difficult patient encounters (DPEs), characterized by interpersonal or communication challenges, are common in clinical medicine and are a potential barrier to optimal patient outcomes and mutual satisfaction amongst patients, families, and physicians [1-2]. These encounters represent 15%-20% of adult outpatient encounters [1]. Internal medicine physicians with fewer years of practice tend to report higher percentages of encounters as difficult [1], suggesting resident trainees may also experience a high frequency of difficult encounters. The frequency of DPEs has not been well described in pediatrics.

Difficult patient encounters are often multifactorial, influenced by: 1) patient and family factors, 2) physician factors, and 3) situational factors [3]. Examples include demanding or nonadherent patients [4], strong emotions, complex medical problems, physician stress or poor communication skills, and time constraints [3]. Challenges may also be intensified by complex family dynamics [5], as pediatricians often communicate with multiple caregivers. Pediatricians may also need to mediate decisional discord between the child and parent [6]. In addition, there may be discordant opinions between health professionals that may further challenge communication between providers and families.

Expert recommendations exist for addressing DPEs in pediatrics. Breuner and Moreno offer strategies to navigate these encounters [5]. Sisk et al. outline models for mediating decisional discord between parents and their children [6]. Platt and Gordon published a book entitled *Field Guide to the Difficult Patient Interview* describing strategies for addressing difficult encounters in detail [7]. However, without formal training, physicians likely learn to handle these encounters through trial and error in high-stakes clinical settings.

This cross-sectional survey served as a needs assessment for curricular development and examines the frequency, clinical contexts, and training preferences related to DPEs. 

Conceptual framework

We utilized the six step approach to curriculum development described by Kern et al. to inform our study, with a specific focus on steps 1 (problem identification and general needs assessment) and 2 (targeted needs assessment) [8].

Findings from this needs assessment survey will be used to inform and modify our program’s existing communication skills training (CST) to specifically address DPEs. Survey items regarding training preferences take into consideration educational methods supported by learning theory relevant to CST (simulation, clinical practice, role modeling, reflective practice, discussion, and lecture) [9-10]. The CST curricula can range from general communication skills to specialized skills (such as genetic counseling), depending upon the program’s needs [9-13], and can improve physicians' communication skills when training includes standardized patient (SP) encounters and role play [14]. In the survey, we explored resident attitudes toward elements of successful CST courses. This was an important goal of our needs assessment survey, given the potential for improvement in patient satisfaction after physician participation in a formal CST course [15].

Gap addressed in this study

To successfully equip pediatric residents with communication skills to navigate DPEs, we needed to identify the contexts that challenge them. Therefore, we sought to further describe pediatric resident experiences with difficult encounters, specifically: 1) frequency of DPEs, 2) self-efficacy in managing these encounters, 3) satisfaction with specific challenging situations, 4) anxiety level with these specific contexts, and 5) self-identified training needs and preferences. We describe our needs assessment for developing a communications skills curriculum addressing DPEs and propose educational strategies to consider.

## Materials and methods

Research design, participants, and setting

This study uses a cross-sectional survey research design with mixed-methods to characterize resident experiences with and training needs for addressing DPEs as a part of a programmatic needs assessment.

Our three year categorical pediatric residency trains 12 residents per class. The residency program is based at a free-standing children’s hospital with 259 beds. For their inpatient clinical experience, residents rotate on the general pediatrics inpatient service for 6-12 weeks during each year of training. For their longitudinal general pediatrics ambulatory experiences, they are scheduled weekly in a longitudinal outpatient "continuity clinic" per Accreditation Council for Graduate Medical Education (ACGME) requirements for pediatric residency training programs in the United States. On the inpatient service, first year residents generally provide direct patient care for four to seven patients each day, while second and third year residents serve in a supervisory role on the inpatient teams. In the outpatient continuity clinic, residents are given 40-minute appointment slots. Both inpatient and outpatient patient loads have been deliberately chosen to allow sufficient time for establishing rapport and adequately addressing the concerns of families of children cared for in our clinical settings. Given our program is based at a tertiary care center, our patients often have complex medical needs. Residents do receive training in communication skills as a part of their existing curriculum (outlined in Table 1), and our needs assessment aimed to outline goals for expanding this curriculum to deliberately cultivate skills for addressing DPEs.

**Table 1:** Residency program curriculum for physician-patient communication skills. SP, standardized patient.

Learning activity	Participating trainees
Reflective group discussion with structured observation of role model videos demonstrating two challenging patient interviews	First, second, and third year residents
Interprofessional SP encounters focusing on leadership and conflict resolution	First and second year residents
SP encounter disclosing a medical error	First year residents
SP encounter responding to a request for stimulants for performance enhancement	First year residents
SP encounter responding to vaccine refusal	First year residents
SP encounter disclosing a positive newborn screen	First year residents
SP encounter using an interpreter	First year residents
SP encounter discussing sexuality	Second year residents
SP encounter discussing refusal of newborn screen	Second year residents
Role play exercises in negotiation	Third year residents

This study was approved by the Johns Hopkins Medicine Institutional Review Board (IRB00072269).

Survey development and administration

We developed the survey tool using an iterative process. The initial version of the survey consisted of one short answer, nine multiple choice, and four Likert-style questions. Several self-efficacy questions were modeled after those used by Hernandez et al. for characterizing resident confidence and satisfaction in encounters with families with limited English proficiency [16]. We then pilot tested the initial version with 17 residents and revised the content based on initial feedback. Revisions included: 1) asking residents to rate confidence, anxiety level and satisfaction with specific difficult encounter types, whereas the original survey only asked about experiences with DPEs in general, 2) standardizing Likert-style questions to all use five-point scales, whereas the original survey used a combination of four-point and five-point scales, and 3) including questions about training preferences that were not asked in the original survey. To inquire about experiences with more specific DPE types, in the revised survey, we introduced six clinical encounter types often described as “difficult,” chosen after review of the literature and discussion with faculty preceptors in our inpatient and outpatient settings (Table 2). Following the revision of the survey to include these additional elements, one author (KC) performed cognitive review with two junior faculty members who were not on the study team. The full survey is given in Appendix A and consisted of three short answers, nine multiple choices, and 27 Likert-style questions. 

**Table 2:** Difficult encounter types included in the needs assessment survey.

Encounter types
Chronic pain: patients with unrelenting chronic pain
Nonadherence: lack of patient compliance with agreed upon treatment plan
Plan insistence: parents demanding a plan that trainee as treating physician is not comfortable with
Bad news: situations in which trainee and their team must deliver difficult life-altering news
Unfocused: parental historian communicating in an unfocused manner, engaging in frequent tangential conversation
Angry: patient or parent who is upset and confrontational

We then pilot tested the survey a second time to determine feasibility of having residents complete a web-based survey sent by email, and based on low-response rate (13/36, 36%), we revise our survey administration method and gave residents protected time to complete a paper survey in order to improve the response rate. 

We distributed the survey at resident educational sessions at the end of the 2016-2017 academic year to new interns during orientation, as well as residents transitioning from first to second year and from second to third year. Verbal consent was obtained at the time of survey administration. Survey responses were anonymous. We offered no incentives for survey completion.

Analysis

We analyzed the results of Likert-style questions using descriptive statistics. The p-value calculations were done using the Fisher’s exact test using GraphPad online software (GraphPad Sofware Inc., San Diego, CA). Box and whisker plots were created using Excel 2016 (Microsoft Corporation, Redmond, WA). Two authors (AH and KC) independently reviewed open-ended responses and coded them to group those with similar content together into categories. In order to triangulate the data, we sent themes from the qualitative analysis back to the residents in order to ensure that they accurately reflected the residents’ viewpoints.

## Results

Thirty-three of 36 total residents in the program responded to the survey, including 12 incoming first year residents, 11 residents transitioning to second year, and 10 residents transitioning to third year, for a response rate of 92% (33/36). Three residents were not present at the educational sessions and were not given the opportunity to complete the survey as we would not be able to ensure the anonymity of their responses.

Attitudes and perceptions

Residents reported the frequency of encounters in both the outpatient and inpatient setting that they perceived as difficult (Table 3). One resident chose not to respond to the question about the frequency of difficult encounters in the inpatient setting. The majority of study participants reported less than 30% of encounters to be difficult in both the outpatient and inpatient settings. Forty-eight percent of residents reported a difficult encounter frequency of less than 10% in the outpatient setting (16/33), and only three percent reported this low frequency in the inpatient setting (1/32). The difference in perceived frequency of difficult encounters in outpatient and inpatient settings was statistically significant (*p* < 0.001). Furthermore, no residents reported the frequency of DPEs as more than 50% in the outpatient setting, but three residents did report this high frequency of DPEs in the inpatient setting (3/32, 9%). 

**Table 3:** Difficult encounters frequency by setting as reported by residents.

Percentage of encounters reported by residents as difficult	Number of residents reporting this frequency of difficult encounters in the outpatient setting (*n* = 33)	Number of residents reporting this frequency of difficult encounters in the inpatient setting (*n* = 32)
<10%	16 (48%)	1 (3%)
10%-30%	14 (42%)	22 (69%)
31%-50%	3 (9%)	3 (9%)
51%-75%	0 (0%)	3 (9%)
>75%	0 (0%)	0 (0%)

Residents were also asked to rate their communication skills with difficult encounters on a five-point Likert scale (poor to excellent, see Appendix A). A majority of residents (70%, 23/33) described their skills as either fair or good. Eight residents (24%) described their skills as very good or excellent.

**Figure 1:**
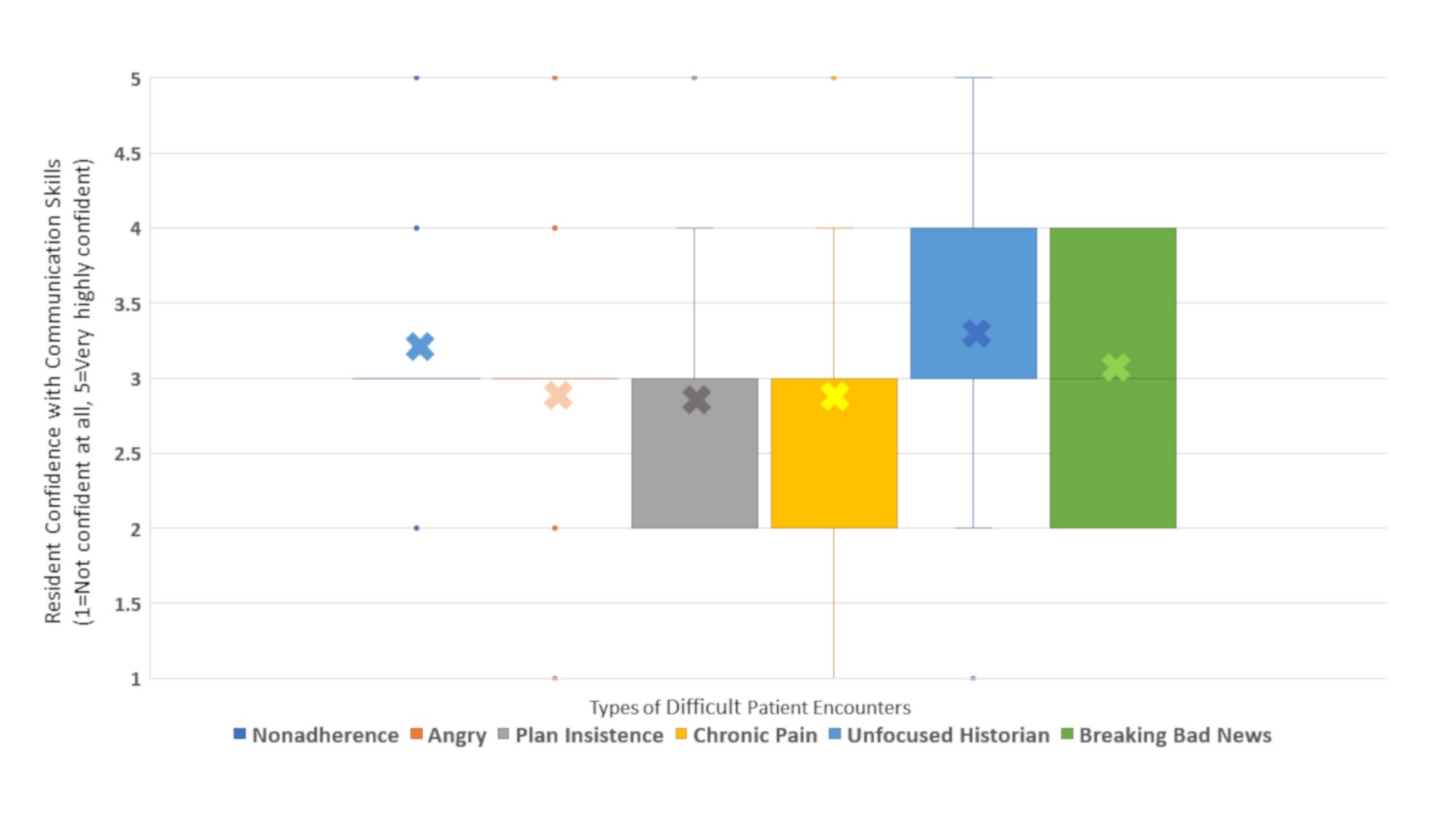
Resident confidence with communication skills in various difficult encounter types.

Residents self-assessed their skills in managing specific DPEs on a five-point Likert scale (very low confidence to very high confidence, see Appendix A). Box plots in Figure 1 display residents’ confidence ratings by difficult encounter type. Residents’ confidence with their communication skills for managing nonadherence and angry families were clustered at the median, whereas the responses to other encounter types had a wider spread. More residents reported low levels of confidence for chronic pain, plan insistence, and breaking bad news when compared with other encounter types.

**Figure 2:**
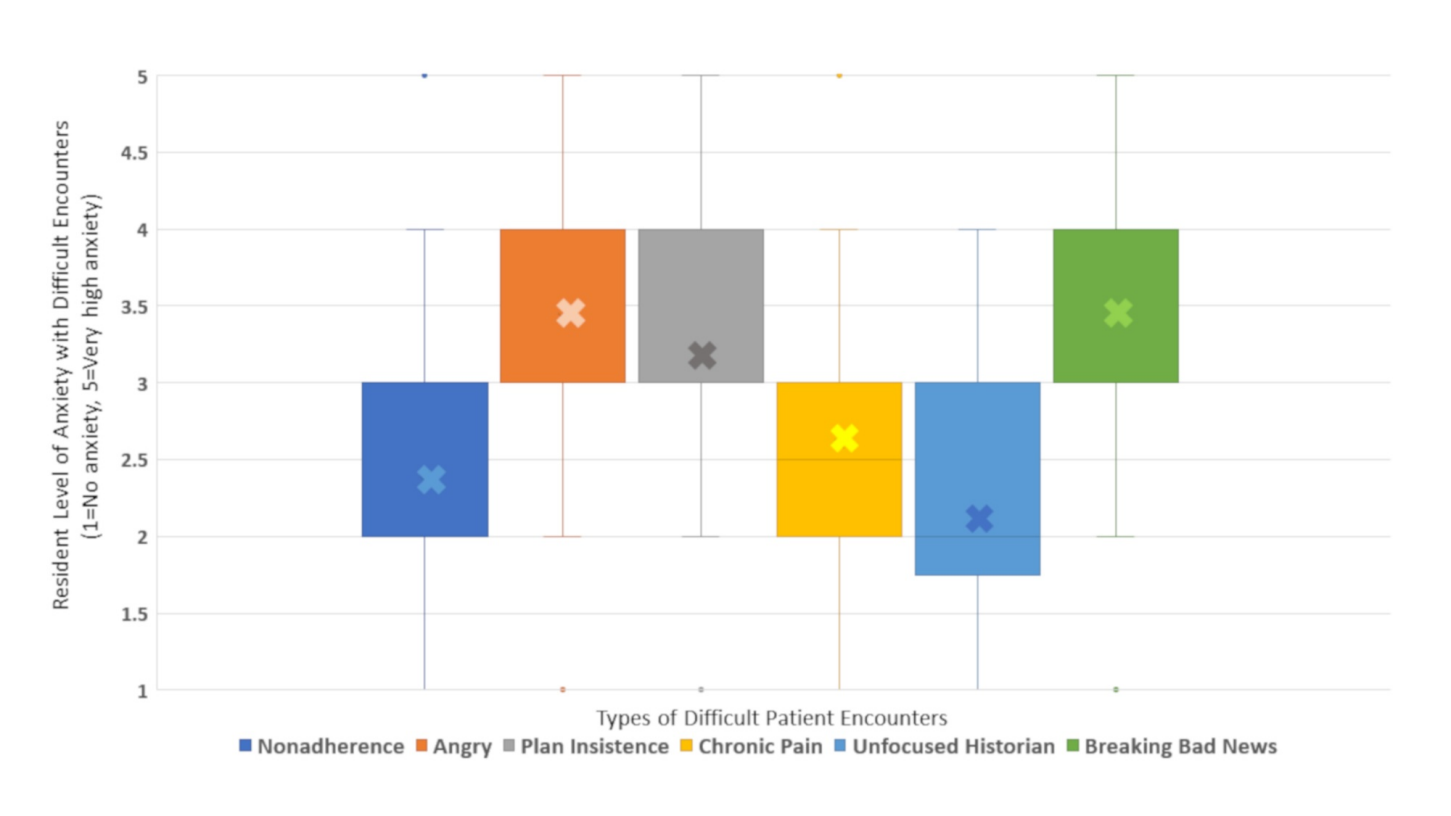
Resident anxiety with various difficult encounter types.

Additionally, residents were asked to consider their level of anxiety with common difficult clinical contexts (Figure 2). Residents generally reported higher levels of anxiety for encounter types involving angry patients and families, families insisting on a plan of care, and when breaking bad news. They noted the lowest levels of anxiety for situations with an unfocused parental historian and patient nonadherence, both of which had median anxiety ratings of 2 (mild anxiety).

**Figure 3:**
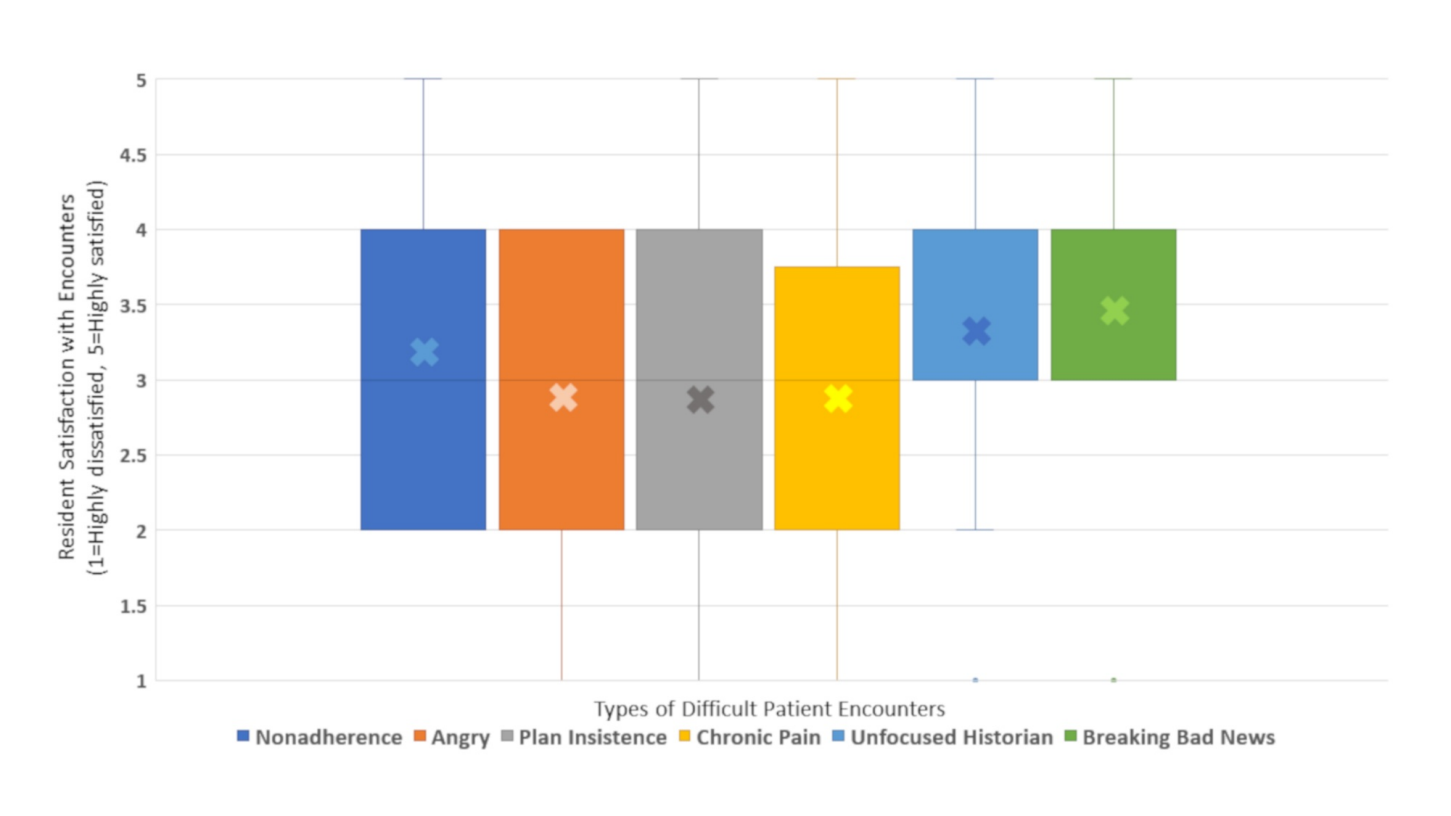
Resident satisfaction in various difficult encounter types.

Residents divulged their level of satisfaction with DPE types as shown in Figure 3. Satisfaction level was modest for all DPE types, with median satisfaction scores of three (neutral) for all scenarios except communicating with an unfocused historian, which had a median score of four (satisfied). However, more residents described being dissatisfied or highly dissatisfied with plan insistence (39%, 13/33), angry caregivers (33%, 11/33), chronic pain (30%, 10/33), and nonadherence (27%, 9/33) than with unfocused historians (18%, 6/33) or breaking bad news (3%, 1/33).

Residents were tasked with rating the importance (not important to essential) of various educational strategies in enhancing their skills for addressing difficult encounters on a five-point Likert scale (see Appendix A). Residents reported the most valuable strategies to be observing role models [*m* = 4.46 (SD 0.95)] and engaging in DPEs in the clinical setting [*m* = 4.84 (SD 0.35)]. Of note, didactic lectures were considered to be the least valuable [*m* = 2.21 (SD 0.97)]. The value ascribed to using simulation to train communication skills for DPEs varied [*m* = 3.63 (SD 1.23)] with 21% of residents (7/33) sharing that this method had no importance or low importance.

In addition, the survey included an open-ended question regarding “what they would like to receive more of” in order to enhance their communication skills for DPEs. Two residents specifically commented that they perceived the training provided in the residency program’s existing communication skills curriculum to be sufficient.

Many residents did report specific ideas for resources and experiences they would like to see included in their training, which are categorized in Table 4. Of note, although many residents did request more practice in the simulated setting, others emphasized a preference for practice in the clinical setting over simulation. One resident requested “*real-life (not practiced in sim) feedback after encounters*,” and another responded that they wanted “*more patient encounters*.” Other residents commented that they needed “*less attendings stepping in to ‘help.*’” Similarly, an incoming intern shared that they would benefit from “*watching role models in difficult encounters (because as med students we were often told to stay out of the room in these situations).*”

**Table 4:** Resources and experiences residents report as necessary to enhance their skills in managing difficult patient encounters.

Resources	Experiences
Communication tips and techniques	Observe role models in clinical settings
Scripts and script training	Simulation and/or role playing
Video examples of effective and ineffective communication	Huddle before anticipated difficult encounters
Cultural competency training	Opportunity to lead difficult conversations
Mindful medicine resources	Deliberate observed practice with constructive feedback

Two open-ended questions elicited the challenges residents experienced with DPEs, which are categorized in Table 5.

**Table 5:** Themes from free response questions regarding characteristics and situations residents cite as challenges.

Disagreement regarding diagnosis or care	Strong emotions	Situational characteristics	Disease-specific factors
Families refusing advised medical care	Patients or families who display anger and hostility	Time constraints	Patients with chronic pain
Families who fail to adhere to treatment plan	Patients or families who display defensiveness	Discussions with adolescents	Patients with vague symptoms
Families insisting on an unadvised care plan	Patients or families who display impatience	Patients who are difficult to discharge	Patients with unexplained symptoms or diagnoses
Families holding unreasonable expectations		Physician's discomfort when patient satisfaction and best practices are at odds	Patients with somatic or factitious disorders
Families in denial about the illness		Delivery of bad or life altering news	Patients who have been abused or neglected
Families who trust inaccurate information		Disclosure of medical errors	Patients who use and abuse narcotics
Families who distrust the medical team		End-of-life discussions	
Family members who disagree with one another			
Disagreement amongst medical team regarding diagnosis or care			

## Discussion

Our study supports the findings of previous studies that DPEs are common and cause distress in physicians [2], and it is the first to our knowledge to describe this finding in pediatric residents. The residents in our study cited many of the same challenges with patient encounters reported in the literature, including responding to demanding or angry patients, as well as treating patients with vague symptoms and chronic pain. It was not surprising to find that residents also expressed relatively low levels of confidence and satisfaction with these encounters. The results from our study contribute further to the literature by the contexts described in responses to open-ended questions by study participants. Another interesting novel finding was our residents’ report of higher frequency of DPEs during their inpatient experiences as compared to their outpatient experiences. Furthermore, this study provides a description of the types of educational methods considered effective by trainees for learning this skill set.

Pediatric residents generally perceived a higher frequency of difficult encounters in the inpatient setting compared to outpatient. Many factors may contribute, including the concentrated time residents spend on inpatient rotations, as well as the complexity of patients admitted to the hospital, as patients with lower acuity and complexity can often be safely managed in the outpatient setting. Children with complex chronic conditions represent an increasing percentage of hospital admissions (10% in 2006), hospital days and resource utilization [17], which may increase the cognitive, technical, and emotional demands of all staff, including residents. Pediatric residents cite challenges of caring for children with medical complexity, including lack of care coordination, complex technology management, patients’ pervasive psychosocial needs and a lack of effective healthcare provider training [18]. Furthermore, the inpatient team is generally responsible for patients’ care from admission to discharge, regardless of the duration and regardless of interpersonal conflict and provider and/or patient dissatisfaction with the communication or relationship. This dissatisfaction may lead to provider avoidance of difficult families and situations, given findings of one study which revealed decreased nursing and physician engagement with difficult families as compared to cooperative families [19]. Enhancing provider confidence and skills for these challenging encounters may help counteract a tendency to avoid challenging families of hospitalized patients. Educational activities in a resident curriculum for addressing difficult encounters should include special attention to the challenges in the inpatient setting.

Many residents reported high levels of anxiety and low levels of confidence in their communication skills for breaking bad news. Interestingly, although residents in our program experienced relatively high levels of anxiety when breaking bad news, they seldom reported breaking bad news to be dissatisfying. This reported anxiety is consistent with findings from a study that described increased indices of physiologic stress as well as self-reported stress on the State-Trait Anxiety Index when delivering bad news compared to taking a routine patient history [20]. Breaking bad news curricula may be well received by residents, and studies suggest this to be a teachable skill [21]. Existing curricula for difficult conversations, such as the American Academy of Pediatrics Resilience in the Face of Grief and Loss Curriculum [22] may aid in this training. Using open-ended responses, our study also identified specific challenges in disclosing a medical error and having conversations at the end-of-life, which may also be important topics to incorporate into future curricula.

Many residents also reported high levels of anxiety when communicating with angry patients and families and addressing families who insist on a plan with which they (as the patient’s primary provider) do not agree. Residents reported wide variation in level of satisfaction with most difficult encounter types, with the exception of encounters with unfocused parental historians or breaking bad news, which less than 25% found to be dissatisfying or highly dissatisfying. Dissatisfaction and anxiety are important to note, as research has shown that this can affect provider wellness, with physicians experiencing a range of emotional and physical reactions, including abdominal pain, exhaustion and fear after they have denied a patient’s request [23]. Furthermore, ambivalence and decisional conflict can cause psychological discomfort [24]. The stress associated with these challenging encounters can not only affect physician wellness, but can also impact decision-making, leading individuals to take more mental shortcuts [25]. A difficult encounters curriculum might also include coping strategies for residents, both to maintain wellness and clarity of thought in the face of anxiety-provoking emotional exchanges. Example activities may include role play or simulation with emotional debrief, small group discussions of challenging experiences (such as Balint groups), or narrative medicine workshops to process emotions and experiences while building empathy. Strategies for mindfulness and empathy can also be taught outside of the clinical setting for residents to incorporate into their daily practice. One example is the BREATHE OUT technique, which includes a structured pre-visit intervention with the mnemonic BREATHE to prompt 1) reflection on provider's Bias or assumption about the patient, 2) reflection upon why patient is difficult, 3) goals to accomplish during the visit, and 4) pause before entering the patient room, as well as a post-visit intervention mnemonic OUT to prompt 1) reflection on the outcome, 2) consideration regarding whether anything unexpected was learned, and 3) anticipation of what the provider would look forward to addressing tomorrow. This BREATHE OUT technique has been shown to improve physician satisfaction with DPEs [26]. These techniques may support residents’ resilience on an individual level, although systems interventions to optimize the learning and working environment, which go beyond the scope of a difficult encounters curriculum, remain important steps to reducing resident anxiety and burnout [27].

Results from the survey identified resident perceptions of strategies that help them learn skills for addressing DPEs. These included simulation, the replication of an experience or event using role play, deliberate observation of role models skillfully engaging in challenging encounters, and group discussions. Interestingly, about 20% of our residents placed relatively lower importance on using simulation as a modality. This may be due to individual learner preferences or resident perception that they already participate in sufficient simulated encounters. Many of the residents surveyed had experienced SP simulations in the past, and it is possible that they felt uncomfortable or did not notice any subsequent improvement in their performance. Furthermore, although learner feedback from the residency's simulation programs has generally been positive, faculty facilitators have varied degrees of training and experience in pre-briefing, facilitation and debriefing, which could have impacted the residents' perception of this training modality.

Of note, learners in our study placed a very high importance on participating in the conversations that take place during difficult clinical encounters and expressed a need to receive more feedback on their communication strategies in these scenarios. The resident request for “*less attendings stepping in to ‘help*’” noted in the free response section suggests that some residents want to be granted greater autonomy to more fully engage in these challenging conversations. Trainees may enter residency without having had much opportunity to lead or even observe challenging conversations, as was suggested by one incoming intern who stated that they would benefit from “*watching role models in difficult encounters (because as med students we were often told to stay out of the room in these situations)*.” Faculty development is important for both providing trainees with feedback and making decisions about entrustability, especially in the context of competency-based medical education [28]. Developing a program where faculty directly observe and coach a group of residents and provide feedback has been shown to improve the resident perception of feedback quality; however, this may be somewhat time intensive, as one successful program required about 10% salary support for each faculty member coaching 10 residents [29]. Faculty development to support learner experience in leading challenging discussions, with support, coaching, debriefing and effective feedback, may be a key to supporting learners in navigating difficult conversations in their clinical practice.

This study has several limitations, including the small number of residents from a single pediatric residency program, therefore limiting the generalizability of responses. Second, residents were asked to estimate the frequency of difficult encounters at a single time point and their responses may be subject to recall bias. Furthermore, some of the residents had responded to a similar survey during the pilot phase which may have influenced their previous reflection on the topic and subsequent responses. A final limitation is that all residents surveyed receive all of their inpatient training at free-standing referral center, which may limit the generalizability to programs where inpatient training occurs in other settings. 

For further study, it would be helpful to have supervising physician and family perspectives on the necessary skill set and training needs for DPEs. Next steps also include development of a curriculum to enhance resident competence in DPEs, which includes training residents in mindfulness techniques and communication skills. As with all effective curricula, faculty development is critical to ensure that supervising attendings can balance role modeling with adequate resident autonomy, as well as provide effective feedback and coaching for DPEs.

## Conclusions

Difficult patient encounters are frequent experiences during pediatric residency training, and trainees value active learning strategies to develop skills to successfully navigate these encounters while maintaining personal job satisfaction and wellness. Although the majority of residents endorsed simulation as an important strategy for training skills for DPEs, it is important to consider blended learning approach with multiple educational modalities, given approximately 20% of residents ascribed low value to simulation. As a program, our next steps include creating a “Difficult Patient Encounters” communication skills curriculum informed by this needs assessment, aimed to enhance patient care as well as raise resident self-efficacy and resilience for these stressful and challenging encounters. In addition to curriculum development for residents, it may be helpful to implement faculty development focused on techniques for supervising resident-led difficult conversations and provide effective debriefing and feedback to promote resident growth.

## Appendices

Appendix A - Survey of resident experience with difficult encounters and communication skills training

Your completion of this survey or questionnaire will serve as your consent to be in this research study.

We are interested in your experience as a trainee with providing care for patients and families in encounters traditionally described as “difficult.”

This includes, but it not limited to: patients with strong emotions, discordance between provider and family regarding diagnostic and treatment approach, patients with vague or numerous symptoms, patients with chronic pain, patients or caregivers with underlying psychiatric illness, delivery of life altering news or end of life counseling to a patient or family.

The information you provide in this survey will serve to help guide the training we provide in this area. You may choose to leave any question blank for any reason.

1. What year of residency are you in?

     a. Incoming first year resident

     b. Transitioning first year resident to second year resident

     c. Transitioning second year resident to third year resident

2. To enhance your skills for difficult patient encounters, what, if anything, do you wish you could receive more of?

3. What patient care scenarios or situations, if any, do you find to be the most challenging or difficult to address?

4. What characteristics, if any, that you have observed in patients and their family members have you found to be the most challenging or difficult to address?

5. For increasing your overall competence (knowledge, skills, and attitudes) with difficult encounters, what importance would you give to the following educational opportunities? (1=Not important at all, 2=Low importance, 3=Moderate importance, 4=High importance, 5=Essential or critical importance?

     Lecture (e.g. powerpoint, chalk talk) ___

     Discussion groups (e.g. small group discussion, case discussion) ___

     Observing role models (e.g. viewing videos demonstrating skills, watching team members have difficult conversations with families) ___

     Simulation (e.g. participating in standardized patient scenarios) ___

     Clinical encounters (e.g. having difficult conversations with your patients) ___

     Reflective practice (e.g. thinking about encounters, journaling, discussing experiences with mentors) ___

6. In terms of your overall training, how important do you think educational sessions regarding difficult patient encounters are?

     a. not important at all

     b. low importance

     c. moderate importance

     d. high importance

     e. essential/critical importance

7. Thinking back to your last inpatient pediatrics rotation, please estimate how often you incorporated these skills into ANY encounter? (1=Never, 2=Less than once a month, 3=Between once a month and once a week, 4=More than once a week but not daily, 5=Daily, 6=Multiple times per day. If you are unaware of this skill, please choose "Never")

     Negotiate an agenda for the visit with parent ___

     Redirect using summary statements and transition statements ___

     Name the patient or parent's emotion ___

     Elicit the patient's perspective ___

     Name shared goals and perspectives ___

     Engage in shared decision making ___

8. What percentage of the encounters with patients or families have you experienced so far in your pediatric residency training would you characterize as "difficult" in the GENERAL PEDIATRIC OUTPATIENT setting? (For incoming residents, please answer based on your general pediatrics experiences in medical school).

     a. <10%

     b. 10%-30%

     c. 31%-50%

     d. 51%-75%

     e. >75%

     f. I have never taken care of a patient during a difficult encounter

9. What percentage of the encounters with patients or families have you experienced so far in your pediatric residency training would you characterize as "difficult" in the GENERAL PEDIATRIC INPATIENT setting? (For incoming residents, please answer based on your general pediatrics experiences in medical school).

     a. <10%

     b. 10%-30%

     c. 31%-50%

     d. 51%-75%

     e. >75%

     f. I have never taken care of a patient during a difficult encounter

10. How would you characterize your communication skills when involved in a difficult encounter?

     a. Poor

     b. Fair

     c. Good

     d. Very Good

     e. Excellent

     f. I have never taken care of a patient during a difficult encounter

11. How confident are you in your communication effectiveness in a recent encounter involving:

(1=Very low confidence, 2=Low confidence, 3=Moderate confidence, 4=High confidence, 5=Very high confidence, N/A=never had an encounter of this type)

     Chronic pain ___

     Nonadherence to treatment ___

     Parental insistence on a plan you are not comfortable with ___

     Delivering bad news ___

     Historian unfocused, prone to tangents ___

     Angry patient or parent ___

The following set of questions may be considered more personal to some people. The responses are improtant to guide our training and assess its effectiveness. However, you may choose to leave any question blank for any reason.

12. Rate how you felt after a recent encounter involving:

(1=Highly dissatisfied, 2=Dissatisfied, 3=Neutral, 4=Satisfied, 5=Highly satisfied, N/A=never had an encounter of this type)

     Chronic pain ___

     Nonadherence to treatment ___

     Parental insistence on a plan you are not comfortable with ___

     Delivering bad news ___

     Historian unfocused, prone to tangents ___

     Angry patient or parent ___

13. How much anxiety did you experience in a recent encounter involving:

(1=No anxiety, 2=Mild anxiety, 3=Moderate anxiety, 4=High anxiety, 5=Very high anxiety, N/A=never had an encounter of this type)

     Chronic pain ___

     Nonadherence to treatment ___

     Parental insistence on a plan you are not comfortable with ___

     Delivering bad news ___

     Historian unfocused, prone to tangents ___

     Angry patient or parent ___

14. How anxious do you become during difficult patient encounters in general?

     a. Very highly anxious

     b. Highly anxious

     c. Moderately anxious

     d. Mildly anxious

     e. Not anxious at all

     f. I have never taken care of a patient during a difficult encounter
